# Supplementary material for: Stress-responsive FKBP51 regulates AKT2-AS160 signaling and metabolic function
Source: Nat Commun. 2017 Nov 23;8:1725. doi: 10.1038/s41467-017-01783-y (PMC5700978; doi:10.1038/s41467-017-01783-y)
Supplement: Supplementary file 1 — Supplementary Information [file 41467_2017_1783_MOESM1_ESM.pdf]

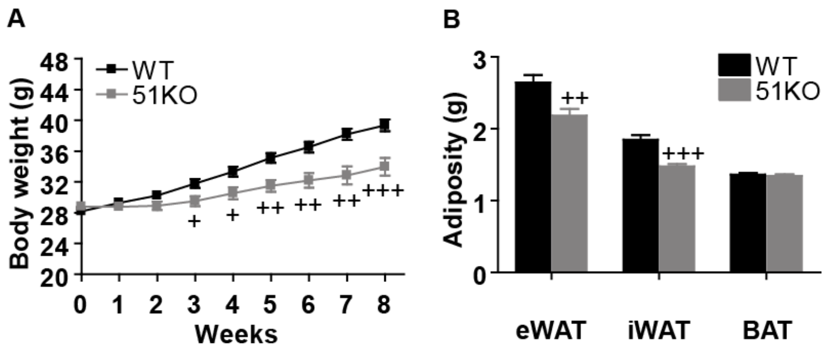

Supplementary Figure 1. Genetic ablation of FKBP51 protects against diet-induced obesity under thermoneutral conditions.

(A) Over 8 weeks of HFD exposure at 30 °C, 51KO mice resisted HFD-induced weight gain and (B) adiposity, indicating that genetic ablation of FKBP51 protects against diet-induced obesity under thermoneutral conditions. n = 8 per genotype group. Repeated measures ANOVA for panel A, 2-tailed t tests for panel B. Data are expressed as means ± SEM. $^{+}P < 0.05$, $^{++}P < 0.01$, $^{+++}P < 0.001$; + significant genotype effect.

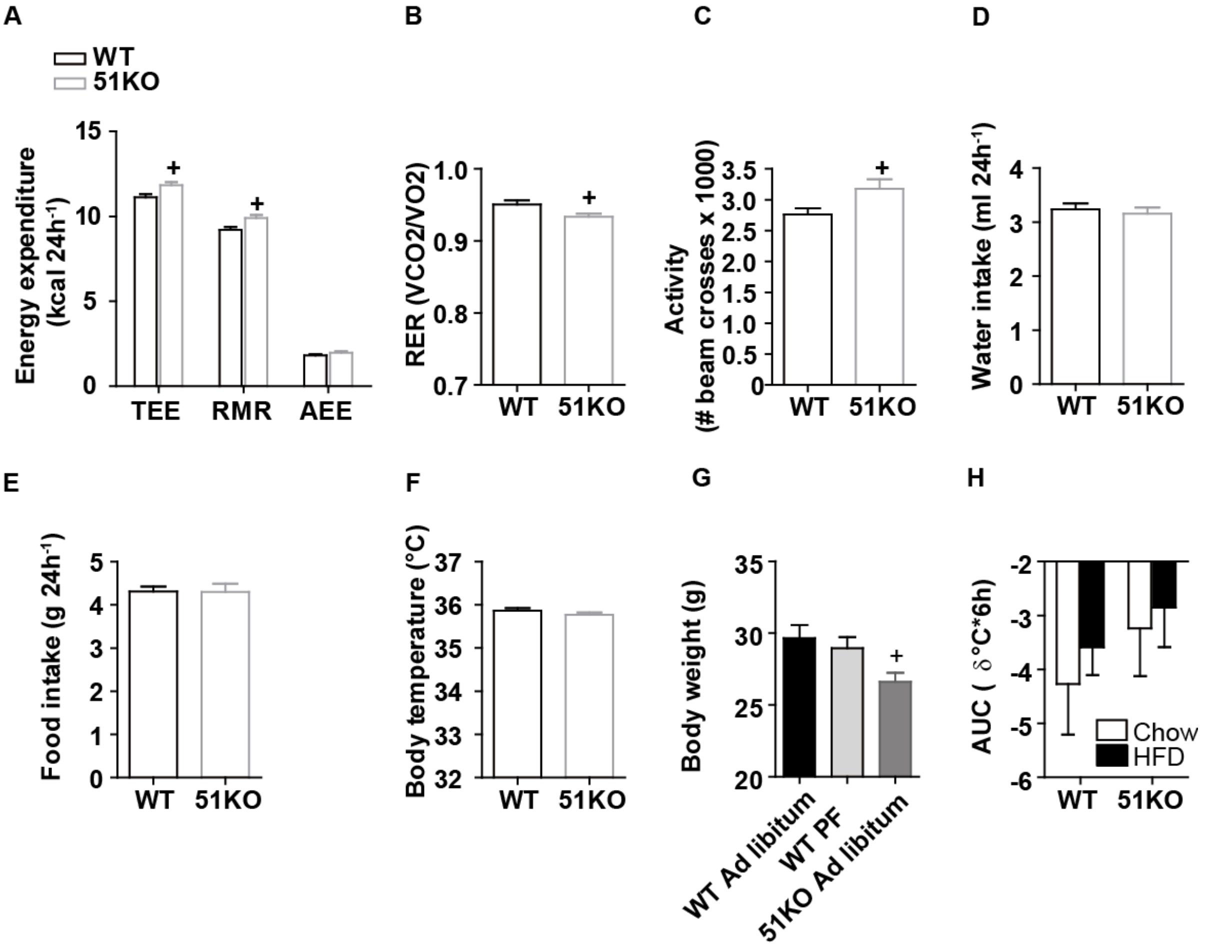

Supplementary Figure 2. Metabolic phenotype in 51KO transgenic mice. Metabolic readouts were assessed in WT mice (n = 18) and 51KO mice (n = 16) under chow conditions in TSE PhenoMaster Systems. (A) Total energy expenditure (TEE), adjusted for body weight, was higher in the chow-fed 51KO animals measured across 24 h. Decomposition of TEE into its resting (RMR) and activity-related (AEE) components revealed that the observed TEE difference was due to increased RMR in the 51KO animals measured over 24 h. AEE did not differ between genotypes as assessed across 24 h. (B) Loss of FKBP51 furthermore decreased the average respiratory exchange ratio (RER) and (C) increased the average home-cage activity assessed across 24 h. There was however no effect of genotype on either (D) water consumption, (E) food intake or (F) body temperature assessed across 24 h. (G) In a separate cohort of animals, a pair-feeding experiment was performed to assess genotype-dependent effects on food intake. 51KO (n = 9) and WT (n = 11) mice in the ad libitum groups had free access to food, whereas WT PF (n = 9) mice were pair-fed to the 51KO mice. After 6 weeks on the pair-feeding paradigm, 51KO mice weighed significantly less compared to WT ad libitum mice. (H) A separate

cohort of mice was examined for cold-induced rectal temperature changes following exposure to 4 °C under control and HFD conditions (n = 11 per group). Neither genotype nor diet had an effect on body temperature following short-term (6 h) cold exposure. Data are expressed as means ± SEM. $^{+}P < 0.05$, ANCOVA adjusted body weight for panels A – C, 2-tailed t tests for panels D – F & H, 1-way ANOVA plus Bonferroni testing for panel G; + significant genotype effect.

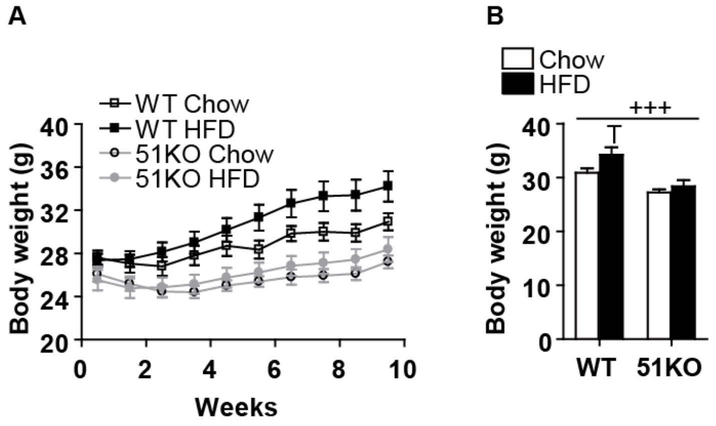

Supplementary Figure 3. Genetic ablation of FKBP51 prevents HFD-induced weight gain observed across 2 independent experiments. In an independent experiment used to assess glucose and insulin tolerance, 51KO mice presented the same body weight phenotype compared to WT mice as reported in Experiment 1. (A) 51KO mice weighed significantly less than WT mice throughout the 8 week dietary treatment and (B) at the experimental end. Furthermore, a tendency was observed indicating that WT mice were susceptible to HFD-induced weight gain compared to 51KO mice as interpreted from body weight progression. Data are expressed as means ± SEM.

$^{T}P < 0.1$, $^{+++}P < 0.001$; + significant genotype effect; T trend for diet, Repeated measure ANOVA for panel A, 2-way ANOVA plus Bonferroni testing for panel B.

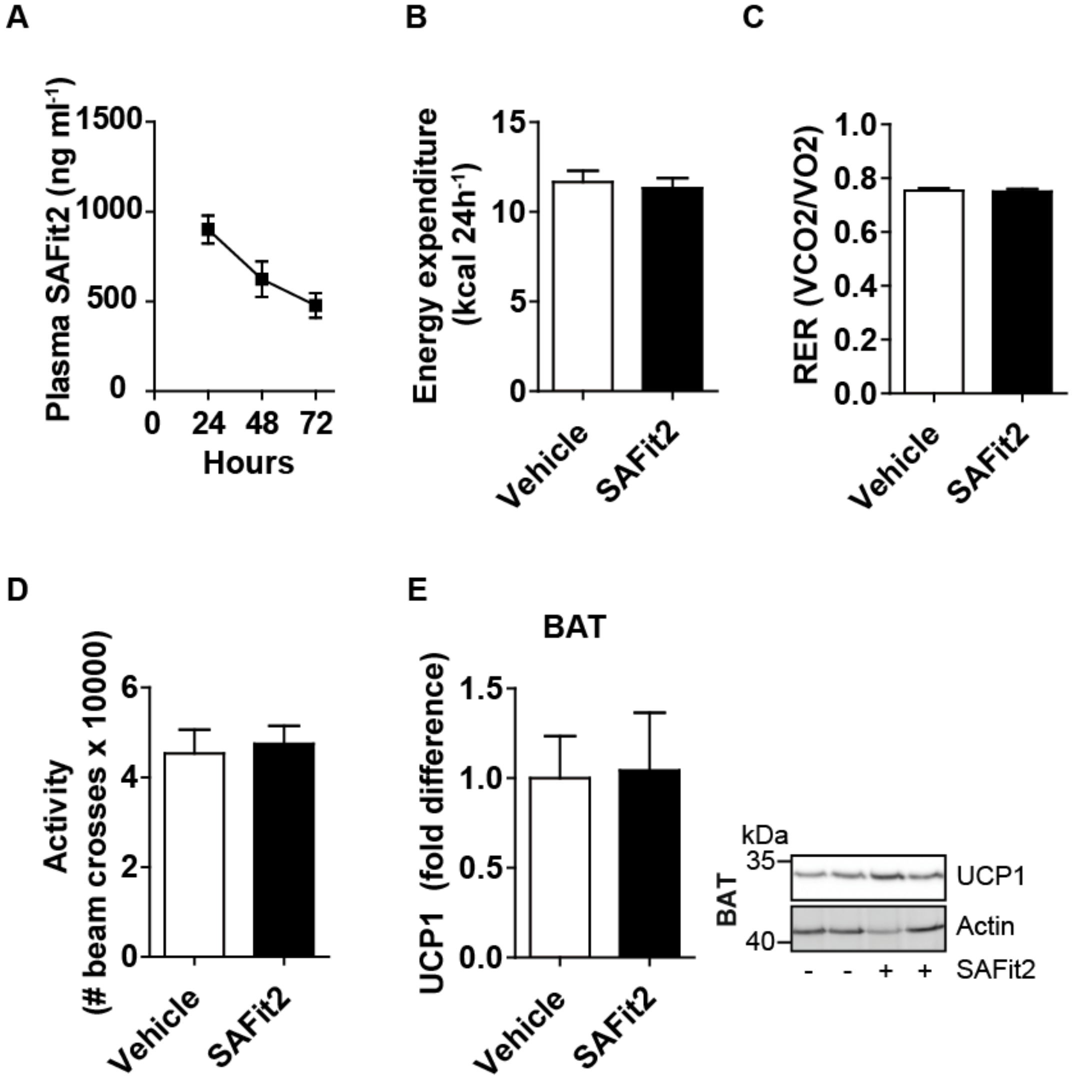

Supplementary Figure 4. Acute administration of the FKBP51 antagonist SAFit2. (A) A single application of slow-release formulated SAFit2 gel (2 mg, s.c.) resulted in high SAFit2 plasma levels measured at 24h, 48h, and 72h post-injection. (B) (B – D) Total energy expenditure (TEE) adjusted for body weight (B), respiratory exchange ratio (RER) (C), and home-cage activity (D) were unaffected by FKBP51 antagonism. (F) Acute SAFit2 had no effect on UCP1 expression in BAT measured 48 h after SAFit2 administration. Panel A: n = 6; panel B – E: n = 8 per treatment group. Data are expressed as means ± SEM. 2-tailed T-tests for panels B – E.

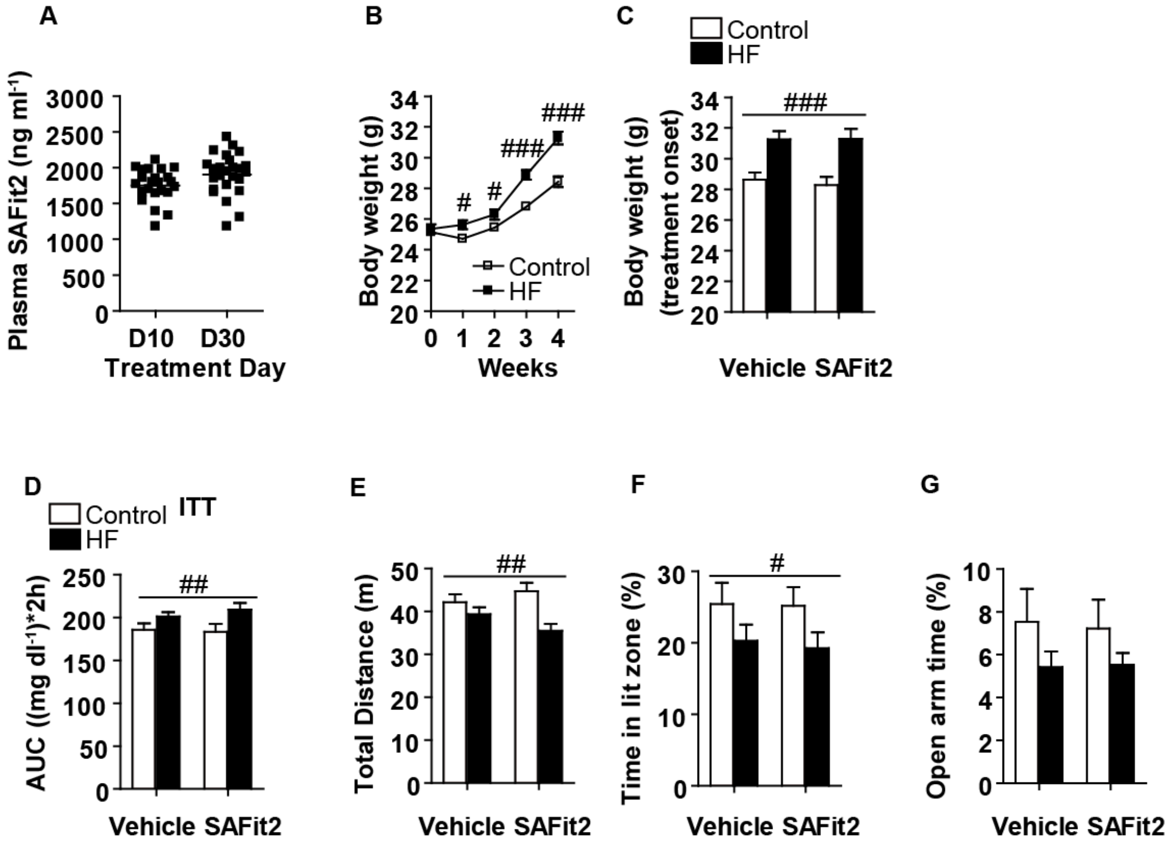

Supplementary Figure 5. Repeated administration of the FKBP51 antagonist SAFit2. (A) Administration of SAFit2 (twice daily at 20 mg per kg) by intraperitoneal injections resulted in high SAFit2 plasma levels and minimal inter-animal variability on day 10 and day 30 of treatment schedule (n = 24 plasma samples for each time point). (B) 4 weeks before SAFit2 treatment onset, mice were randomly assigned to either the control diet or HFD group. Exposure to the HFD resulted in a significant increase in body weight progression (n = 25 per diet group) (C) At treatment onset, mice were subdivided into treatment groups counterbalanced by body weight, and therefore within each dietary group there was no difference in body weight. (D) Although insulin tolerance was significantly impaired by HFD exposure, there was no improvement on account of SAFit2 treatment, demonstrated by the glucose AUC. (E) SAFit2 treatment had no effect on locomotor activity assessed as total distance traveled in an open field, although HFD exposure significantly reduced locomotor activity. (F – G) 30-day administration of SAFit2 had no undesirable behavioral side effects on anxiety-like behavior examined in both the dark-light transition test and the elevated plus maze test. By contrast, exposure to a HFD decreased the time spent in the lit compartment of the dark-light test. Panels A – E: n = 12 Vehicle-Control, n = 13 SAFit2-

Control, n = 12 Vehicle-HFD, n = 13 SAFit2-HFD). Data are expressed as means ± SEM. $^{\#}P < 0.05$, $^{\#\#}P < 0.01$, $^{\#\#\#}P < 0.001$, Repeated measures ANOVA for panel B, 2-way ANOVA for panels C – G; # significant diet effect.

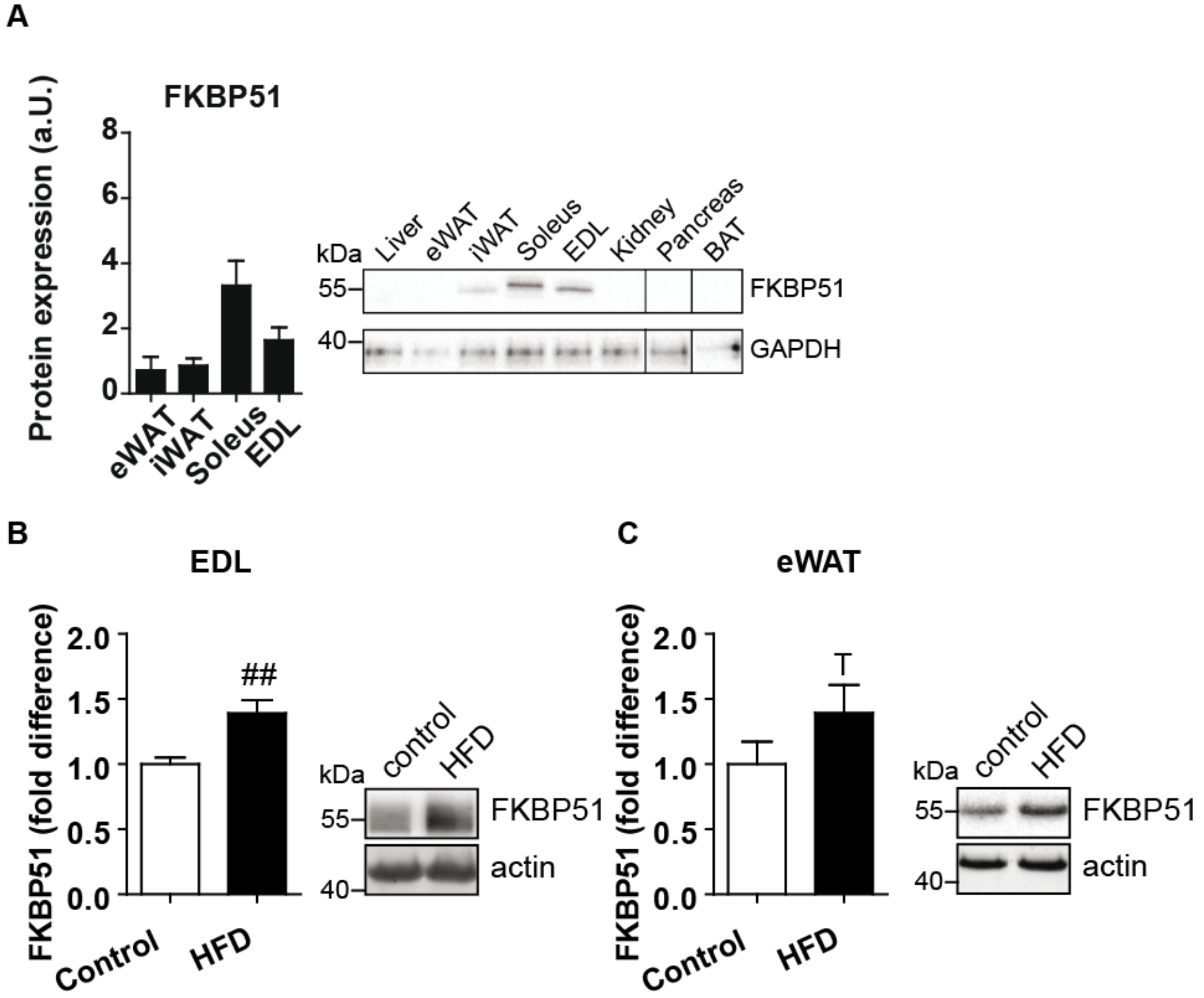

Supplementary Figure 6. Molecular characterization of FKBP51. (A) FKBP51 protein expression was detectable in eWAT, iWAT, soleus muscle, and EDL muscle, whereas was not detected in liver, kidney, spleen, pancreas, gut, or BAT. For FKBP51 tissue expression, n = 6 per tissue. (B) 8 weeks of HFD exposure significantly increased FKBP51 protein expression in EDL skeletal muscle, n = 6 per group. (C) A tendency for HFD-induced FKBP51 expression was furthermore detected in eWAT, n = 6 per group. Data are expressed as relative fold change compared to control diet condition ± SEM. $^{\#\#}P < 0.01$, 2-tailed t tests for panels B – C; # significant diet effect, T trend for diet.

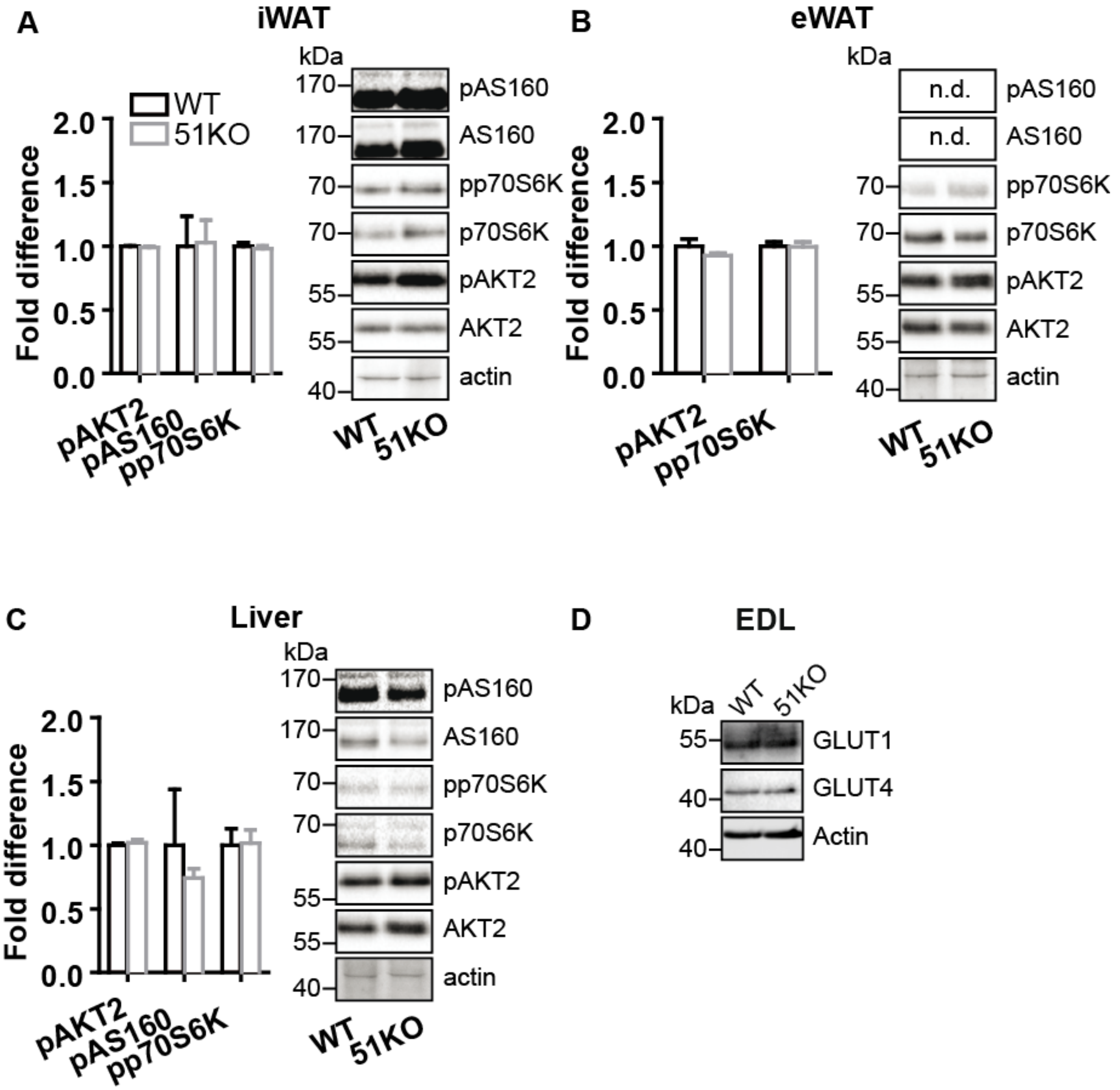

Supplementary Figure 7. FKBP51 selectively effects the insulin signaling pathway and glucose uptake. Insulin signalling assessed by pAKT2, pAS160, and pp70S6K protein expression was not affected by FKBP51 genetic ablation in iWAT (A), eWAT (B), or liver (C). (D) Total GLUT1 and GLUT4 expression EDL muscle was unaffected by FKBP51 deletion. For quantification of phosphorylated protein, n = 4 per genotype. For GLUT1/4 expression in EDL muscle n = 3 per group. Data are expressed as relative fold change compared to wild-type condition ± SEM. 2-tailed t tests for panels A – C. ‘n.d.’ denotes ‘not detectable’.

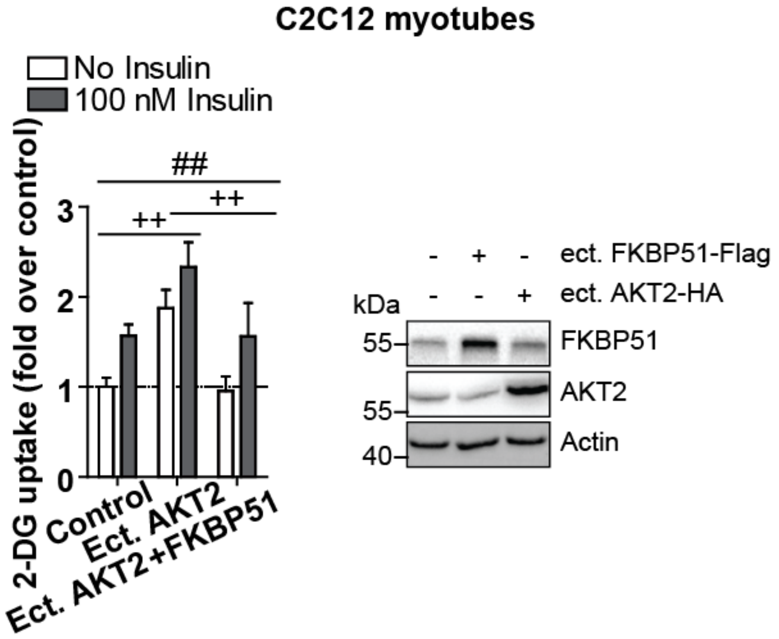

Supplementary Figure 8. Effects of ectopic overexpression of FKBP51 and AKT2 on glucose uptake. In C2C12 myotubes, enhanced glucose uptake from AKT2 overexpression was lost by simultaneous FKBP51 overexpression. Confirmation of ectopic overexpression in differentiated C2C12 cells harvested 4 days post-transfection assessed by FLAG-tagged FKBP51 and HA-tagged AKT2 antibodies. Data are expressed as relative fold change compared to control condition ± SEM. $^{++}P < 0.01$, $^{##}P < 0.01$, 2-way ANOVA plus Bonferroni testing; + significant genotype effect, # significant insulin effect.

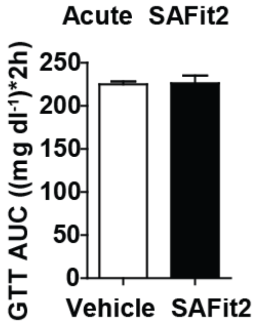

Supplementary Figure 9. Effects of FKBP51 antagonism on 6h glucose tolerance

Acute administration of SAFit2 (20mg per kg, i.p.) had no effect on glucose tolerance measured 6h following SAFit2 delivery. n = 12/treatment. Data are expressed as means ± SEM. 2-tailed T-tests.

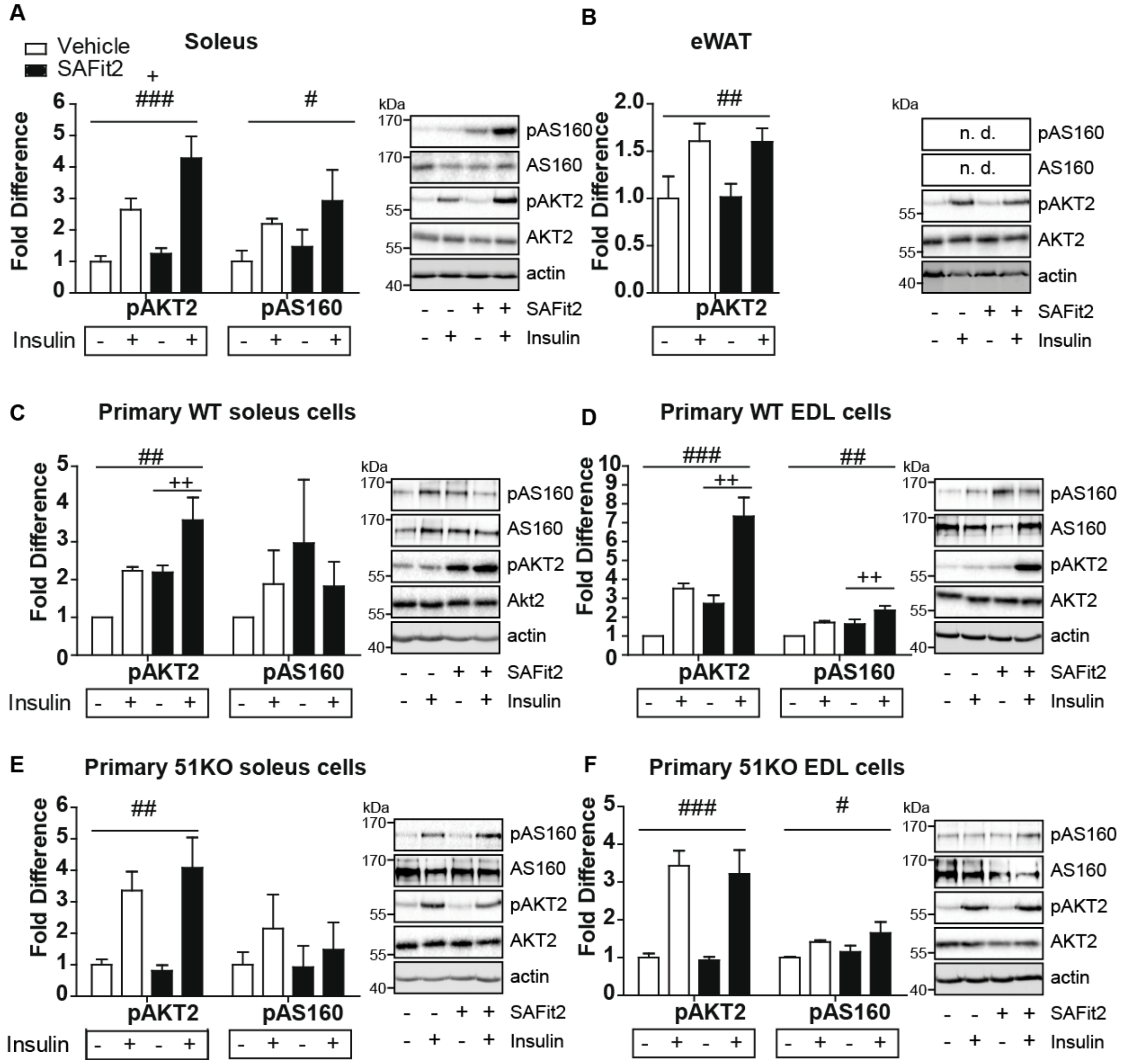

Supplementary Figure 10. Effects of FKBP51 antagonism on insulin signaling pathway and glucose uptake. (A) SAFit2 treatment increases pAKT2 in soleus muscle and insulin increases both pAKT2 and pAS160 in soleus muscle. (B) SAFit2 has no effect on pAKT2 in eWAT, whereas insulin increased pAKT2 expression. (C – D) SAFit2 increased pAKT2 in primary EDL and soleus myotubes from WT mice, and also increased pAS160 in EDL myotubes. (E – F) By contrast, there was no effect of SAFit2 treatment in cells harvested from 51KO skeletal muscle (EDL or soleus). Data are expressed as relative fold change compared to control condition ± SEM. For quantification of phosphorylated protein expression in mice, n = 6 per group and in primary myotubes, n = 3 per group. $^{+}P < 0.05$, $^{++}P < 0.01$, $^{\#}P < 0.05$, $^{\#\#}P < 0.01$, $^{\#\#\#}P < 0.001$. 2-way ANOVA for panels A – F; + significant treatment effect, # significant insulin effect.

A

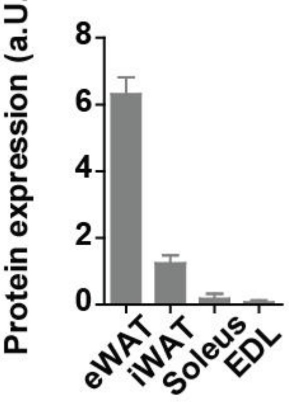
Protein expression (a.U.)
8
6
4
2
0
eWAT
iWAT
Soleus
EDL

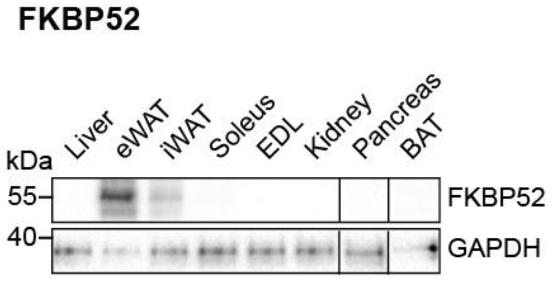
FKBP52
kDa
Liver
eWAT
iWAT
Soleus
EDL
Kidney
Pancreas
BAT
55
40
FKBP52
GAPDH

B

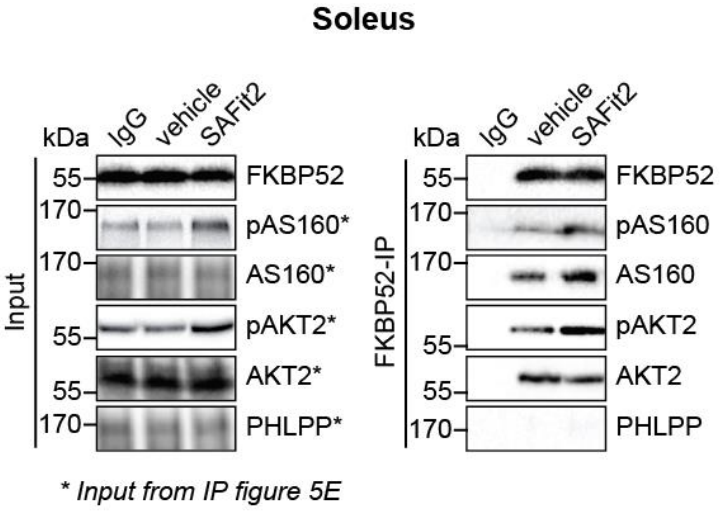
Soleus
kDa
IgG
vehicle
SAFit2
Input
55
170
170
55
55
170
FKBP52
pAS160*
AS160*
pAKT2*
AKT2*
PHLPP*
FKBP52-IP
55
170
170
55
55
170
FKBP52
pAS160
AS160
pAKT2
AKT2
PHLPP
* Input from IP figure 5E

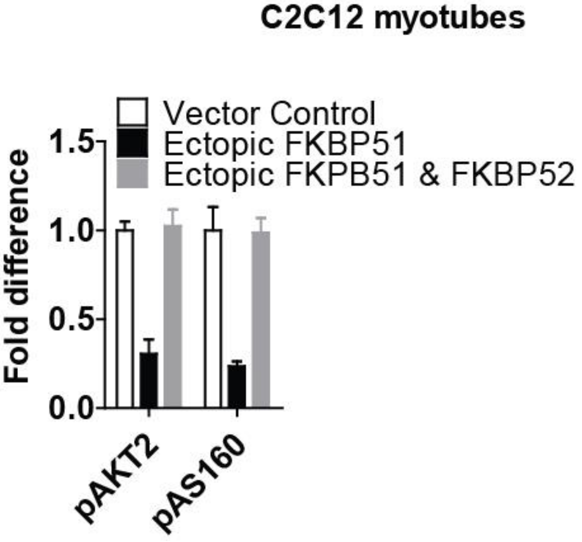
C2C12 myotubes
Vector Control
Ectopic FKBP51
Ectopic FKPB51 & FKBP52
Fold difference
1.5
1.0
0.5
0.0
pAKT2
pAS160

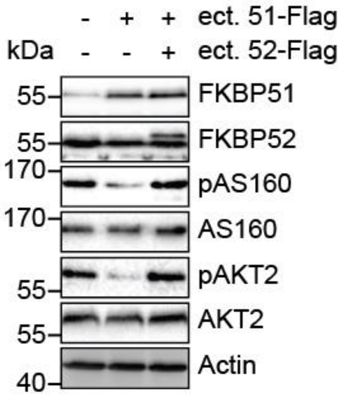
- + + ect. 51-Flag
kDa - - + ect. 52-Flag
55
55
170
170
55
55
40
FKBP51
FKBP52
pAS160
AS160
pAKT2
AKT2
Actin

Supplementary Figure 11. FKBP52 expression and function account for muscle-specific effects of FKBP51 loss and antagonism. (A) Quantified FKBP52 protein expression in eWAT, iWAT, soleus muscle, and EDL muscle, n = 6 per tissue. (B) Tissue lysates from 30-day vehicle-treated or SAFit2-treated mice exposed to HFD were immunoprecipitated with anti-FKBP52 and then analyzed by Western blot using FKBP52, (p)AKT2, (p)AS160, and PHLPP. Immunoprecipitation reactions revealed that FKBP52 is in complex with AKT2 and AS160 but not PHLPP. (C) Ectopic overexpression of FKBP51 in C2C12 myotubes decreased pAKT2 and pAS160 expression. By contrast, simultaneous ectopic overexpression of FKBP51 and FKBP52 prevented the FKBP51-dependent decreases in expression of pAKT2 and pAS160. Panel A n = 6 per tissue; panel B n = 3 per group; panel C n = 4 per group. Data are expressed as relative fold change compared to control condition ± SEM. 'a.U.' denotes arbitrary units.

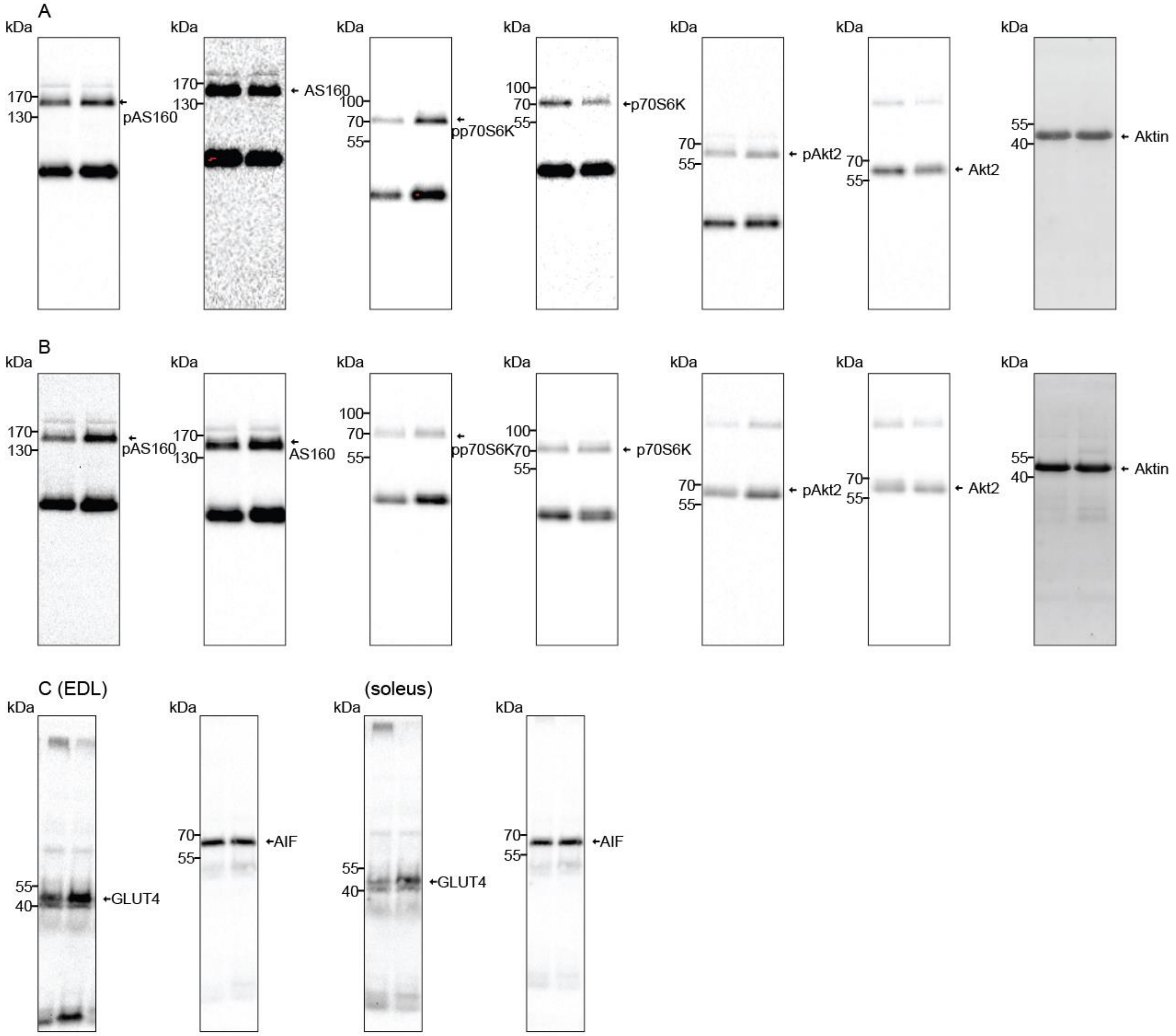

Supplementary Figure 12. Uncropped Images of Western blots from Figure 4. Uncropped Western Blots from Fig. 4A (A), Fig. 4B (B), Fig. 4C (C)

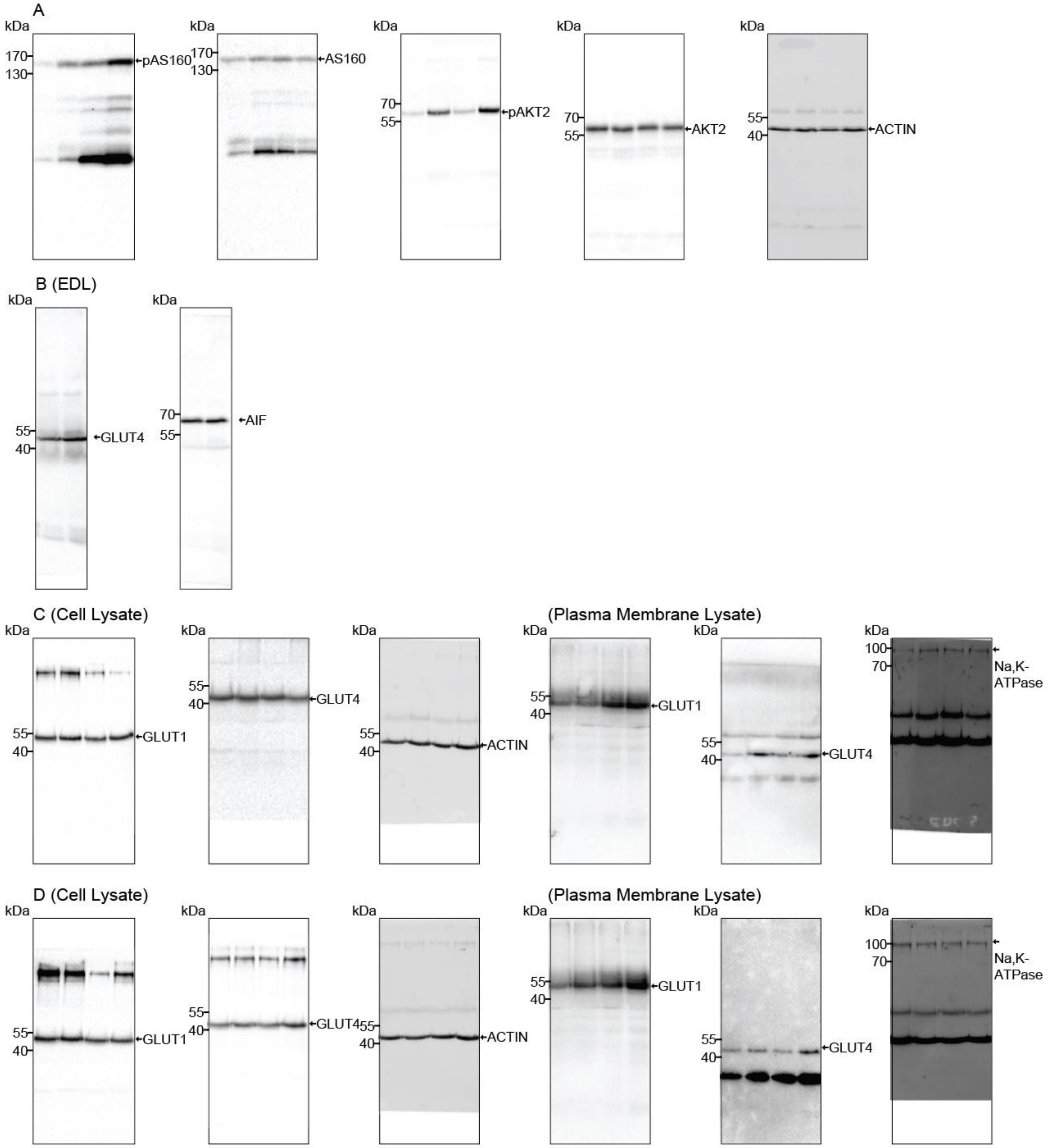

Supplementary Figure 13. Uncropped Images of Western blots from Figure 5. Uncropped Western Blots from Fig. 5A (A), Fig. 5B (B), Fig. 5C (C), Fig. 5(D)

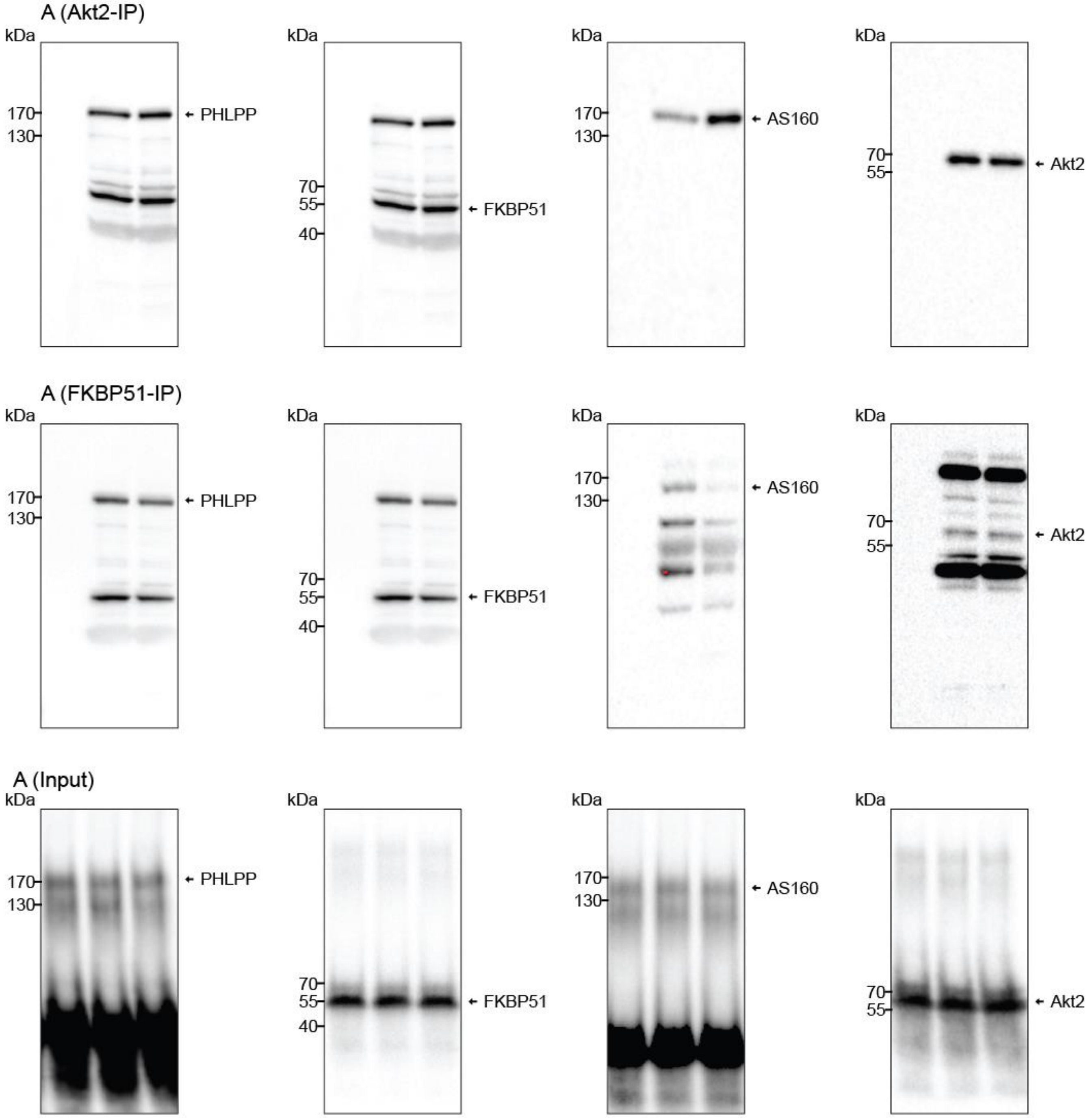

Supplementary Figure 14. Uncropped Images of Western blots from Figure 6. Uncropped Western Blots from Fig. 6A (A)

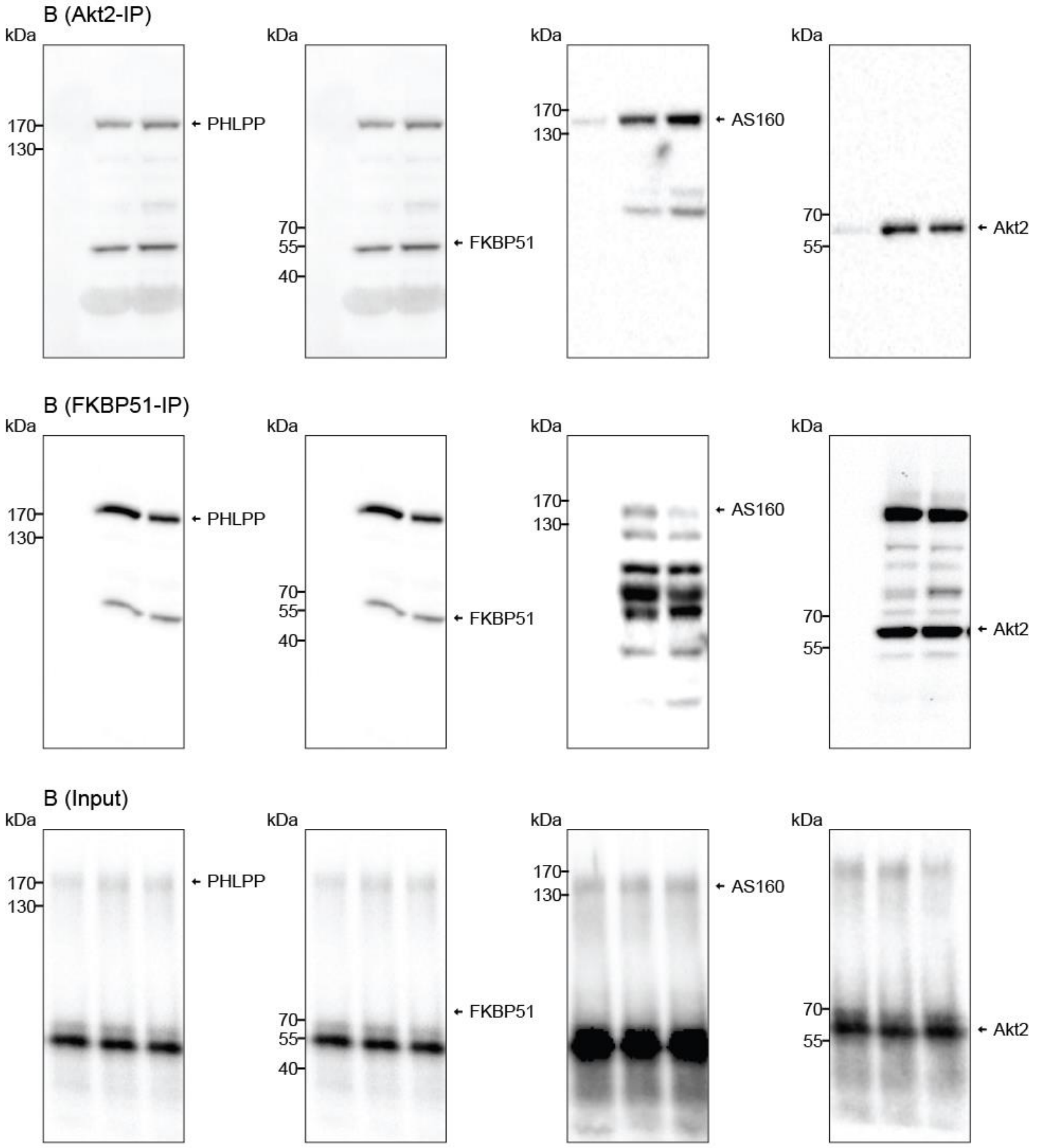

Supplementary Figure 15. Uncropped Images of Western blots from Figure 6. Uncropped Western Blots from Fig. 6B (B)

Supplementary Methods

Indirect Calorimetry

Energy expenditure was assessed using indirect calorimetry (TSE PhenoMaster, TSE Systems, Bad Homburg, Germany). Briefly, animals were allowed to habituate to the indirect calorimetry cages for 48h before data were collected. Following 48 h of acclimatization, O2 consumption and CO2 production were measured every 5 min for a total of 68.5 h. Indirect calorimetry was performed at room temperature (experiment 1, cohort 1). Heat production (referred to as total energy expenditure (TEE), [kcal 24_h^{-1}]) was calculated from O2 consumption (VO2, [ml h^{-1}]) and CO2 production (VCO2, [ml h^{-1}]) using the Weir equations [1]. The respiratory exchange ratio (RER) was calculated as the ratio of volumes of CO2 produced to volumes of O2 consumed (VCO2/ VO2). Home-cage locomotor activity was assessed by beam breaks using an ActiMot infrared light beam system within the calorimetry system.

Assessment of Energy Expenditure Components

The delay in acquisition between the indirect calorimetry measurements and the instantaneous locomotor activity measurements was corrected for by a deconvolution procedure, which was performed using a two-compartment gas diffusion model [2,3], taking into account chamber washout characteristics at a cage size of 8 L and a flow rate of 0.45 L min^{-1}. Following deconvolution, total energy expenditure (TEE, kcal $24h^{-1}$) was decomposed into its activity-related energy expenditure (AEE) and resting metabolic rate (RMR). RMR was modelled in a time-dependent manner using a method based on penalized spline regression, allowing for the detection of up to four RMR frequency components per 24 h (8 knots $24h^{-1}$) [2]. Cases were excluded if the correlation between the convoluted activity and TEE did not reach an arbitrary cutoff of $r \geq 0.7$. Model fit was assessed by visual inspection of the component analysis residuals.

Pair-Feeding Paradigm

Mice were initially singly-housed one week prior to the experimental onset. On day one of the pair-feeding paradigm, 51KO mice (n = 9) and WT mice (n = 11) received ad libitum access to HFD (D12331, Research Diets, New Brunswick, NJ, USA). A second group of WT mice (WT-PF) were pair-fed to the 51KO mice. Each

day for 6 weeks, mice in the WT-PF group received restricted access to the HFD, defined as the amount consumed by the 51KO mice 2 days earlier. Food was weighed and replaced daily at 08:00. If residual food remained in the cages of WT-PF mice, it was removed and weighed prior to the delivery of the next daily food portion.

Cold-Induced Thermoregulation

51KO and WT males were divided into a control diet (10.5%kcal fromfat,D12329, ResearchDiets, Inc., New Brunswick, NJ, USA) and a HFD group (n = 11/group). After 5 weeks on their respective diets, cold-induced thermoregulation was monitored. Initially rectal body temperature was measured for 4 days prior to the cold exposure paradigm to habituate mice to the rectal thermocouple probe. On the 5th day, rectal temperatures were monitored at 0, 2, 4, and 6 h following exposure to 4 °C using an Oakton Acorn Temp JKT Thermocouple Thermometer (Oakton Instruments, IL, USA).

Intraperitoneal Glucose Tolerance Test (GTT)

An intraperitoneal injection of D- glucose (2 g per kg body weight) was delivered to fasted mice (14 h fast). Blood glucose levels were measured on blood collected by tail cut at 0, 30, 60, and 120 min intervals following the glucose load. Glucose levels were measured using a handheld Contour XT glucometer (Bayer Health Care, Basel, Switzerland). Plasma was also collected from blood at 0 and 30 min to assess fasting insulin and glucose-stimulated insulin levels, respectively.

Intraperitoneal Insulin Tolerance Test (ITT)

Mice were fasted for ~12 h and subsequently received intraperitoneal injections of 0.5 IU per kg body weight of insulin. Blood glucose levels were assessed from tail cuts at 0, 30, 60, and 120 min following the insulin load. Glucose levels were measured using a handheld Contour XT glucometer (Bayer Health Care, Basel, Switzerland).

Behavioral Analyses

All behavioral tests were performed between 08:00 and 12:00 (Experiment 4). General locomotor activity was examined in an empty open field arena over 15 min under 15 lux illumination. Anxiety-related behaviors were

assessed using the elevated plus maze and dark-light transition tests as previously described [4]. Each test was videotaped by an overhead camera and analyzed using the automated video-tracking software ANYmaze4.9 (Stoelting, Wood Dale, IL, USA).

Hormone Quantification

Plasma insulin levels were determined using a mouse metabolic magnetic bead panel (Millipore Corp. Billerica, MA, USA; sensitivity: insulin 14 pg ml^{-1}). For the assessment of fasting insulin levels, blood was collected 14 h following an overnight fast by tail cut. Similarly, glucose-stimulated insulin levels were measured from plasma taken from blood collected 30 min following glucose load during the glucose tolerance test.

Preparation of vesicular phospholipid gels

Vesicular phospholipid gels (VPGs) with a phospholipid content of 50% (m/m) were prepared as slow releasing formulation for SAFit2. Encapsulation of SAFit2 into the gel was done by a direct incorporation method of the poorly water-soluble substance. Therefore, accurately weighted phospholipid was solved in ethanol (100%) and a stock solution of SAFit2 (20.0mg ml^{-1} in 100% ethanol) was added. Then the solvent was evaporated for two days under constant vacuum in a drying oven (25 °C, 10 mbar). The solid mixture was hydrated with buffer and homogenized by dual asymmetric centrifugation using a Speedmixer™ DAC 150.1 FVZ (Hauschild GmbH & Co KG, Hamm, Germany) for 45 minutes at 3500 rpm.[1,2] The final SAFit2 concentration in the gel was 10mg g^{-1}.

SAFit2 Quantification

The concentration of plasma SAFit2 was quantified following acute administration of the SAFit2 gel and following intraperitoneal administration on treatment days 10 and 30 by Liquid chromatography/mass spectrometry (LC/MS/MS). Briefly, plasma was analyzed using the combined high-performance liquid chromatography/mass spectrometry (HPLC/MS-MS) technique. Analysis was performed using an Agilent 1100 Series (Agilent, Waldbronn, Germany) liquid chromatograph which was interfaced to the ESI source of an Applied Biosystems API 4000 (ABSciex, Darmstadt, Germany) triple quadrupole mass spectrometer. All samples were added to Ostro protein precipitation and phospholipid removal plates (Waters, Eschborn,

Germany). Deuterated clomipramine (Clomi-D3) was used as internal standard. Chromatography was performed using a gradient elution in an Accucore RP-MS 2,6µm column (2.1 x 50 mm, Thermo Scientific, Dreieich, Germany) at a flow rate of 0.3 ml min^{-1} and 30°C.

Toxicity Assays

LDH assay To assess membrane disruption and cell death, the release of lactate dehydrogenase (LDH) into the growth medium of C2C12 cells was measured. The assay (LDH cytotoxicity detection system) was carried out according to the manufacturer's protocol (Clontech, Mountain View, CA, USA). As a positive control, 0.02% Triton X-100 was added to the medium 1-2 h prior to performing the assay; wells containing only medium were used as a negative control.

MTT assay C2C12 cells were incubated in the presence of 0.5 mg mL^{-1} tetrazole 3-(4,5-dimethylthiazol-2-yl-)-2,5-diphenyltetrazolium bromide (MTT) for 4 h at 37°C and 5% CO2. The plates were read as described previously [8].

Western Blot Analysis

To analyze insulin signaling, tissues from 5 h-fasted WT and 51KO mice (n = 4/group) were harvested 5 min after insulin administration (0.75 IU per kg). To analyze FKBP51 expression, various tissues (brown adipose, EDL, eWAT, iWAT, kidney, liver, pancreas, and soleus muscle) were harvested from adult C57BL6/N male mice (n = 6). Tissues were homogenized in lysis buffer containing 62.5 mM Tris-HCl, 2% SDS, and 10% sucrose supplemented with protease (Sigma, P2714) and phosphatase (Roche, 04906837001) inhibitor cocktails, and subsequently centrifuged at 12,000 x g to remove cell debris. Lysates were sonicated three times, and protein concentrations were measured using the BCA assay (BCA Protein Assay Kit, Life Technologies, Darmstadt, Germany). After dilution, protein samples (40 µg) were heated for 5 min at 95°C in loading buffer. Equal amounts of proteins were separated by SDS-PAGE and electro-transferred onto nitrocellulose membranes. Non-specific binding was blocked in Tris-buffered saline, supplemented with 0.05% Tween (P2287; Sigma-Aldrich, St. Louis, MO, USA) and 5% non-fat milk for 1 h at room temperature. Blots were subsequently incubated with primary antibody (diluted in TBS/0.05% Tween) overnight at 4 ºC. A list of primary antibodies used in this study is provided below. The following day, blots were washed and probed with the respective horseradish

peroxidase secondary antibody for 1 h at room temperature. Immuno-reactive bands were visualized either using ECL detection reagent (Millipore, Billerica, MA, USA, WBKL0500) or directly by excitation of the respective fluorophore. Band intensities were evaluated with the ChemiDoc Imaging System (Bio-Rad, Laboratories Inc., Hercules, CA, USA).

List of all primary antibodies:

**Primary Antibody**	**Dilution**	**Phosphorylation site**	**Company**
**actin**	1:5000	/	SCBT, sc-1616
**AKT2**	1:2000	/	CST, #3063
**AS160**	1:1000	/	CST, #2670
**p70S6K**	1:1000	/	CST, #9202
**pAKT2**	1:1000	S473	CST, # 4058
**pAS160**	1:1000	T642	CST, #8881
**pp70S6K**	1:1000	T389	CST, #9234
**AIF**	1:1000	/	Chemicon, #AB16501
**HA**	1:7000	/	Roche, 11667475001
**FLAG**	1:10000	/	Rockland, 600-401-383
**GLUT4**	1:1000	/	CST, #2213
**FKBP51**	1:1000	/	Bethyl, A301-430A
**PHLPP1**	1:1000	/	Millipore, #07-1341
**GLUT1**	1:1000	/	Thermofisher, PA1-1063
**FKBP52**	1:1000	/	Bethyl, A301-427A
**UCP1**	1:1000		Abcam ab10983

Quantification of Protein Data

The level of each phosphorylated protein was normalized to its respective non-phosphorylated protein. For total protein content, actin or AIF were used as internal controls.

Supplementary References

1. WEIR, J. B. New methods for calculating metabolic rate with special reference to protein metabolism. *J.Physiol* **109,** 1–9 (1949).

2. van Klinken, J. B., van den Berg, S. A. A., Havekes, L. M. & Willems Van Dijk, K. Estimation of activity related energy expenditure and resting metabolic rate in freely moving mice from indirect calorimetry data. *PLoS One* **7,** (2012).

3. Arch, J. R., Hislop, D., Wang, S. J. & Speakman, J. R. Some mathematical and technical issues in the measurement and interpretation of open-circuit indirect calorimetry in small animals. *Int.J.Obes.(Lond)* **30,** 1322–1331 (2006).

4. Schmidt, M. V *et al.* Postnatal glucocorticoid excess due to pituitary glucocorticoid receptor deficiency: differential short- and long-term consequences. *Endocrinology* **150,** 2709–2716 (2009).

5. Shefer, G. & Yablonka-Reuveni, Z. Isolation and culture of skeletal muscle myofibers as a means to analyze satellite cells. *Methods Mol. Biol.* **290,** 281–304 (2005).

6. Kim, D. *et al.* A small molecule inhibits Akt through direct binding to Akt and preventing Akt membrane translocation. *J. Biol. Chem.* **291,** 22856 (2016).

7. Gassen, N. C. *et al.* Association of FKBP51 with Priming of Autophagy Pathways and Mediation of Antidepressant Treatment Response: Evidence in Cells, Mice, and Humans. *PLoS Med.* **11,** (2014).

8. Zschocke, J. *et al.* Antidepressant drugs diversely affect autophagy pathways in astrocytes and neurons--dissociation from cholesterol homeostasis. *Neuropsychopharmacology* **36,** 1754–68 (2011).
